# Interoception and the uneasiness of the mind: affect as perceptual style

**DOI:** 10.3389/fpsyg.2015.01408

**Published:** 2015-09-17

**Authors:** Sibylle Petersen, Andreas von Leupoldt, Omer Van den Bergh

**Affiliations:** ^1^Institute for Health and Behaviour, University of Luxembourg, Esch-sur-Alzette, Luxembourg; ^2^Research Group on Health Psychology, KU Leuven, Leuven, Belgium

**Keywords:** interoception, predictive coding, affect, categorization, symptom perception

## Abstract

Autonomous system models of interoception describe perception of bodily sensations as an active process in which the brain generates and tests hypotheses about the body on the basis of proximal information. This view of perception as inference allows a new perspective on the role of affect in perception. Affect and interoception are closely linked, but processes underlying this link are poorly understood. We suggest that a predictive coding perspective allows acknowledging affect as integral part of information processing. We outline how affect may intrinsically modify processes of interoception by acting as threshold mechanism in stimulus grouping and information compression. We outline how well-established methods, for example, from categorization research may allow quantifying this influence of affect on perception in empirical tests of predictive coding models. We discuss how this may enrich the study of the relationship between affect and interoception and may have important clinical relevance.

## Introduction

How accurate are people when they describe their internal states? This is a question of high clinical relevance, especially in a medical context where report of bodily symptoms has important diagnostic and therapeutic implications. Symptoms such as pain or dyspnea cannot be measured by objective means, but only by self-report (Parshall et al., 2012). Unfortunately, we are apparently poor judges of what is going on inside of our body (Wiebe et al., 1994; Janssens et al., 2009; Petersen et al., 2011). Studies estimate that depending on disease domain up to 81.6% of patients report at least one symptom that cannot be related to physiological dysfunction (Hiller et al., 2006). This is frustrating for both, patients and medical doctors, since distress communicated by patients is usually profound and genuine (Brown, 2004; Edwards et al., 2010). On the other hand, a high number of individuals with severe pathology does not report symptoms and does not seek medical help. This can have serious consequences, for example (among other), in the case of stroke (e.g., Lacy et al., 2001), pain (e.g., Yong et al., 2001; Gagliese, 2009), or respiratory disease (e.g., Petersen et al., 2014a).

Anxiety and negative affect are usually treated as prime suspects in research on bias in the perception of bodily sensations. As state and trait, they seem to play a prominent role in the perception of the world within and outside the body (Watson and Pennebaker, 1989; Van den Bergh et al., 2004; Cisler and Koster, 2010). A meta-analysis on attentional bias toward threatening visual stimuli in anxiety reports an aggregated effect size of *d* = 0.45 (Bar-Haim et al., 2007). Effects of anxiety on the perception of heartbeat are even more robust and a review reports a weighted mean effect size of *d* = 0.62 for anxiety sensitivity, indicating that individuals high in fear of bodily sensations are more sensitive to perceive their heartbeat (Domschke et al., 2010). At the same time, negative affect and anxiety are positively related to an interoceptive bias and a tendency for catastrophizing interpretations (Watson and Pennebaker, 1989; Van den Bergh et al., 2004; Leeuw et al., 2007).

Despite robust empirical evidence that anxiety has an effect on perception and interoception, recent reviews agree that there is a problematic lack of theory-driven studies on why and how anxiety affects attention, information processing, and self-report, in other words on how anxiety becomes part of the perceptual process (Bar-Haim et al., 2007; Cisler and Koster, 2010). In the following, we discuss (1) how to define accurate perception, (2) how recently developed theories of perception as hypotheses testing may allow to integrate affect as part of perceptual algorithms, and (3) how these models can be tested with validated research paradigms.

The perception of bodily sensations is a particularly interesting field to conceptualize affect as part of the perceptual process. Bodily sensations interact closely with emotions (Dunn et al., 2010). Furthermore, they are often complex and ambiguous, involving multiple afferent sources (Parshall et al., 2012). They can also be ambiguous because of a low signal to noise ratios (e.g., heartbeat) or because of low perceived predictability (Browning et al., 2015; Seidel et al., 2015). We suggest below that affect is part of the perceptual processes by guiding disambiguation. Thus, ambiguity of bodily sensations make them an example par excellence to outline our ideas. We suggest, however, that the model presented here is not restricted to the perception of the body, but can be applied also to other forms of perception.

## The Quest for Truth in the Perception of Bodily Sensations

Truth is a matter of definition. Different perspectives have been suggested such as *correspondence*, *consensus*, and *pragmatic* truth (Kruglanski, 1989) or *criterion* and *construct* validity (Cronbach and Meehl, 1955). The *correspondence theory of truth* defines accuracy as correlation between a percept and an external criterion. The *consensus theory of truth* regards perception as validated if it is socially shared, for example, if patients agree with other patients or with health care professionals about symptoms and symptom severity. Correspondence and consensus validity are usually low in the perception of bodily sensations (e.g., Macquart-Moulin et al., 1997; Fallone et al., 2004; Hiller et al., 2006; Janssens et al., 2009; Riegel et al., 2010) and this constitutes a problem for the diagnostic process. In a biomedical model, symptoms need to be “proven” by physiological measures.

This emphasis on external criteria contrasts with a ‘pragmatic’ psychophysics perspective (Thurstone, 1927; Killian, 1985), with neural models of information processing (Friston, 2005; Barrett and Simmons, 2015), and with philosophical models of perception (Hume, 1739/2007; James, 1909/1974; Hohwy, 2012). These models agree that the brain cannot access the world—including the peripheral body- directly, but needs to rely on patterns of brain activity (Hohwy, 2012). Working with proximal stimulation, the brain creates a statistical model of the body in the world that allows to make inferences about causes and predictions about future stimulations (Friston, 2005; Hohwy, 2012). In this perspective “reason must be considered as a kind of cause of which truth is the natural effect” (Hume, 1739/2007, p. 121) and not vice versa. The brain relies on *pragmatic* truth which optimizes the “truth’s cash-value in experiential terms” (James, 1909/1974, p. 5). This inference process does not necessarily reach conscious awareness, but is considered an automatic process running on different hierarchical levels of information processing (e.g., Friston, 2005; Edwards et al., 2012).

Perception of bodily sensations can become gradually independent from physiological changes, creating interoceptive “illusions” such as nocebo or placebo effects (Van den Bergh et al., 2002; Büchel et al., 2014; Constantinou et al., 2015). To assume that perception of placebo and nocebo effects is *flawed* is a misconception resulting from confusing cause and effect (Hume, 1739/2007). Despite low correspondence/consensus validity, pragmatic validity can be high (Cronbach and Meehl, 1955) and related to an overall optimization of perception and coping in terms of a cost/benefit strategy (Huttenlocher et al., 2000). Only if statistical models of the body are not updated in response to error feedback, suboptimal models may lead to suboptimal perception and coping, as it has been suggested for medically unexplained symptoms (Brown, 2004).

To summarize, perception is not measurement and the impact of the emotions and contextual variables on perception is *not* measurement error nor a flaw in perception. Keeping perception and perceptual bias context dependent and tuned-in to situational shifts in costs and benefits is an important skill, not a defect (Lynn and Barrett, 2014; Petersen et al., 2015).

## Anxiety as “Uneasiness of the Mind”

Predictive coding models give up the notion of an agent or self on a superordinate level that supervises perception, but conceptualize perception as representation of statistical regularities in stimulation (Hohwy, 2012). This idea can be traced back to Hume (1739/2007) who proposed that the mind constructs perceiver and percept at the same time. If perception is not coordinated by a self, but the self is a consequence of perception, affective states such as anxiety need to be considered not as something a self “has,” but as part of the data processing algorithms. In this process, the *uneasiness of the mind* is a reaction to the challenge to integrate unspecific, disconnected, and often inconsistent experiences. Hume (1739/2007, Book I Part IV) suggests that in response to this challenge “the mind is bound to be uneasy and to seek relief from that uneasiness” by constructing a self that is the illusion of a continuous existence. This self -in an illusion of agency- can feel and try to evade threats to its existence.

Hume’s uneasiness of the mind has been rediscovered by recent theory development. Negative affect has been conceptualized as resulting from a mismatch between predicted stimulation and experienced stimulation (Joffily and Coricelli, 2013) or as a response to reduction of prediction error at a rate that is lower than expected (Van de Cruys, 2014). We suggest that another important way in which anxiety may be inherent to algorithms of data processing is as decision rule for solving the infinity problem (e.g., Chater and Vitányi, 2003). From the limited data provided by our senses, the brain could create an infinite number of patterns of experiences. Thus, a central problem in experience is which rules the brain applies to choose those experiential categories that are most useful in a pragmatic sense, that is, condensed enough to facilitate perception and not too condensed to miss important details (Reed, 1972; Anderson, 1991).

We propose that negative affect may lead to an over-emphasis on simplicity and reduction of redundancy to the point of loss of unique data. This can be adaptive in situations in which fast information processing is crucial. If excessive simplification becomes a habitual processing style, this may lead to serious reduction of predictive power.

More specifically, we propose that negative affect and anxiety impact on perceived homogeneity of sensation categories (Petersen et al., 2014b, 2015). Categories are defined by both (1) a central tendency and (2) dispersion of stimuli around this point. The dispersion determines how much confidence the brain can have in inferences based on a category prototype. Animal studies suggest that confidence estimates (i.e., estimates of stimulus dispersion) do not require meta-cognition (e.g., Hampton, 2001; Foote and Crystal, 2007; Kepecs et al., 2008).

A homogeneous category acts as precise prior, that is, a likelihood distribution with small dispersion. Lower perceived variability within a category may lead to more extensive generalization of information about the category to new stimuli (e.g., Shepard, 1987; Zaman et al., 2015). We suggest that negative affect is related to a tendency to process the broad picture in categorical terms and less on a single stimulus basis (Robinson and Clore, 2002), resulting in more extensive generalization of category-information, and in the case of aversive categories, fear-generalization.

Biological history has shaped the structure and function of the brain to give priority to deriving valence from stimulation, dividing sensations very broadly in “good” or “bad” to make affective predictions (Barrett and Bar, 2009). In the case of aversive interoceptive stimuli such as pain or breathlessness, highly homogeneous categories allow nearly instant predictions and affective evaluations with high confidence. This allows fast activation of coping behavior with little or no sensory analysis (Petersen et al., 2015). Thus, processing data at a highly condensed category level and ignoring seemingly redundant information may provide a subjective feeling of relief, at least in the short-term, because it allows avoidance of direct stimulus processing. It may help to create and defend a feeling of certainty in the face of ambiguous information (Quellar et al., 2006; Petersen et al., 2015). To summarize, we propose that affect does not only guide data collection in terms of quantity, attention, and speed (Thayer and Lane, 2000; Koster et al., 2004; Cisler and Koster, 2010), but that affect sets a threshold for the degree to which information is condensed and how strongly it is edited to fit into categories.

As threshold mechanism, affect may determine the extent to which information is eliminated as redundant or irrelevant and it may determine when information seeking is aborted in favor of a decision based on priors and prototypes. Thus, we propose a modified vigilance avoidance model (e.g., Mogg and Bradley, 1998) in which negative affect is not related to absolute avoidance of stimulation, but to a shift from more detailed processing to highly condensed prototypes. Thus, high processing priority for interoceptive stimuli in more anxious individuals (e.g., Domschke et al., 2010) may not lead to more rigorous testing of incoming sensory stimulation. In threat-sensitive persons negative affective predictions may initiate the application of overly condensed category-related information and more aggressive stimulus disambiguation by using category information. Focus on condensed category prototypes in turn will make detection of details less likely that would be needed to update categories with regard to central tendency and dispersion.

## Taking Inferential Leaps Between Branches of the Perceptual Tree

Predictive coding approaches place a particular emphasis on brain hierarchies involved in building a representation of the world (e.g., Friston, 2005; Edwards et al., 2012). Lower levels of this hierarchy predict basic sensory attributes in the form of fast and automatic inferences, which are usually gated out of awareness (e.g., breathing sensations at rest). More complex inferences are suggested to be passed on to higher brain structures and—passing the gate of conscious processing—implicit priors may become explicit hypotheses (Hohwy, 2012).

Categorization models equally emphasize hierarchy (Kemp et al., 2007). We propose a continuum ranging from sensory grouping to perceptual grouping to explicit categorization. On the lowest level, at the interface between world and senses, anatomy of the sensory system will group stimulation and process it in a limited number of afferent systems. This processing will be further subjected to perceptual grouping (patterns of stimulation in space and time) and categorization at a central level leading to conscious perception of bodily sensations.

**FIGURE 1:**
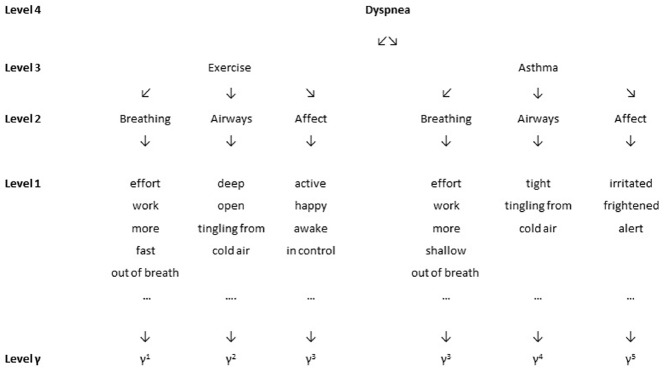
**Level 4 presents the concept dyspnea as superordinate category, Level 3 presents examples of subordinate categories that can define the superordinate category on Level 4.** Level 2 and 1 represent the variability of experience within each subordinate category. Level y presents the level of sensory data. Please note that for the sake of simplicity, we present only three of the multiple experiential categories which may be included in the experience of dyspnea. Please note also that this example represents the experience reported by one single person and is not intended to be a general model of dyspnea perception.

Interoception, just as exteroception, means taking “inductive leaps given very sparse data” (Kemp et al., 2007). Leaps may be made in both directions, from sensation levels to conclusion about categories, or from categories to sensations. Interoceptive categories are seldom fully distinct and misclassification (leaps to the wrong branch) can easily occur. Figure 1 illustrates overlap between interoceptive categories using the example of asthma and sports. Mistaking breathlessness during exercise as a symptom of respiratory disease may lead to exercise avoidance, reduced physical fitness, and disease aggravation (Wanrooij et al., 2014).

Asthma and panic are another example of two distinct categories that overlap strongly at the symptom level. Breathing sensations such as a tight chest and feelings of suffocation occur during asthma and panic attacks (Lehrer et al., 2002). While taking asthma medication (short-acting beta agonists) during an asthma attack alleviates symptoms, sensations induced by this medication such as increased heartbeat and tremor (Salpeter et al., 2004) may aggravate panic. If etiologically distinct symptom categories that require distinct courses of action overlap experientially, inferences based on the “wrong” priors/prototypes may be useless or even harmful. Again, we suggest that this applies to priors on all levels.

## Indices of Perceived Category Homogeneity and Complexity

We propose that affect increases (1) perceived homogeneity of interoceptive categories and confidence in information inferred from these categories, (2) their subjective accentuation, and (3) the impact of these precise priors on perception. Testing dispersion of categories of bodily sensations rather than mean values is a new methodological approach that allows testing innovative hypotheses brought forward by recent predictive coding models. Placing our model in a categorization framework has the advantage that these as yet untested hypotheses can be targeted with established paradigms and statistics developed in the long-standing tradition of categorization research.

To calculate homogeneity of a sensation category, parameters such as standard deviation (SD), coefficient of variation, or range can be computed (Park and Judd, 1990; Boldry et al., 2007; Petersen et al., 2014b). SDs are usually higher for higher means and the coefficient of variation -as the ratio of SD divided by the mean—is recommended if mean values underlying SDs differ widely.

Furthermore, individual slopes across categories allow estimating perceived category variability (for a tutorial on individual slopes analysis, see Pfister et al., 2013). In this analysis, the location of the stimulus on the dimension underlying the category is used to predict, for example, self-report of stimulus intensity or unpleasantness. Steeper slopes indicate stronger differentiation between stimuli (Petersen et al., 2015). Studies are missing that test the effect of trait and state affect on perceived homogeneity within sensation categories, but we expect negative affect and anxiety to be related to lower dispersion (less steep slopes) and to stronger generalization.

Direct similarity ratings or confusion frequencies between stimuli within categories (e.g., Shepard, 1987) allow to compute category homogeneity without explicitly defining dimensions of experience (e.g., Borg and Groenen, 2005; Petersen et al., 2008). Similarity ratings and confusion frequencies can be analyzed in a signal detection approach calculating sensitivity for differences within and between categories and decision criteria. These approaches have been used in studies on interoceptive categorization using respiratory stimuli. Results suggest that individuals high in habitual symptom report and negative affect accentuate stronger between-category differences and show more liberal decision strategies (Petersen et al., 2015).

Furthermore, perceived overlap of sensation categories can be assessed using overlap-indices (Rafaeli-Mor et al., 1999). As an example, we calculated overlap, defined as average communality, for the data given in Figure 1. In this example, one person has selected twelve descriptors to describe dyspnea during “exercise” and 10 descriptors for dyspnea related to “asthma.” Five descriptors were used to describe both situations. Mean overlap is calculated as *OL* = (5/12+5/10)/2 = 0.46 indicating a perceived overlap of 46%. Depression has been suggested to be related to lower complexity in self-perception (Rafaeli-Mor et al., 1999), but we are not aware of research testing the relationship of anxiety, negative affect, or depression with blurred category boundaries in the perception of bodily sensations.

Other analytical strategies to test for differentiation between sensation categories include contingency tables. Taking the example in Figure 1, a 2 × 2 table can be drawn including in its cells the number of attributes that have been assigned only to one of the two categories or are shared by both categories. Chi^2^ for this 2 × 2 table is X^2^ = 4.96, *p* = 0.026 indicating that the attribution of descriptors to the two situations is significantly different from a random distribution. Other indices such as *Cramer’s V* or *Phi* allow to analyze distributions of attributes across contingency tables larger than 2 × 2 (Showers, 1992). They range between 0 and 1 with 1 indicating complete compartmentalization of attributes. In the example of Figure 1, *Cramer’s V* = 0.54, indicating moderate differentiation between exercise and asthma in the choice of descriptors.

## Conclusion

Theories developed over the last centuries are in agreement about the relative and autonomous character of perception. We propose that this new perspective can be fruitfully applied to empirical paradigms using category homogeneity as estimate of confidence of the brain in perceptual inferences and predictions. This perspective may allow testing how affect is inherent to the perceptual process.

### Conflict of Interest Statement

The authors declare that the research was conducted in the absence of any commercial or financial relationships that could be construed as a potential conflict of interest.
